# Influence of Calcium in Extracellular DNA Mediated Bacterial Aggregation and Biofilm Formation

**DOI:** 10.1371/journal.pone.0091935

**Published:** 2014-03-20

**Authors:** Theerthankar Das, Shama Sehar, Leena Koop, Yie Kuan Wong, Safia Ahmed, Khawar Sohail Siddiqui, Mike Manefield

**Affiliations:** 1 Centre for Marine BioInnovation, School of Biotechnology and Biomolecular Sciences, The University of New South Wales, Sydney, Australia; 2 Department of Microbiology, Quaid-i-Azam University, Islamabad, Pakistan; 3 School of Biotechnology and Biomolecular Sciences, The University of New South Wales, Sydney, Australia; INRA Clermont-Ferrand Research Center, France

## Abstract

Calcium (Ca^2+^) has an important structural role in guaranteeing the integrity of the outer lipopolysaccharide layer and cell walls of bacterial cells. Extracellular DNA (eDNA) being part of the slimy matrix produced by bacteria promotes biofilm formation through enhanced structural integrity of the matrix. Here, the concurrent role of Ca^2+^ and eDNA in mediating bacterial aggregation and biofilm formation was studied for the first time using a variety of bacterial strains and the thermodynamics of DNA to Ca^2+^ binding. It was found that the eDNA concentrations under both planktonic and biofilm growth conditions were different among bacterial strains. Whilst Ca^2+^ had no influence on eDNA release, presence of eDNA by itself favours bacterial aggregation via attractive acid-base interactions in addition, its binding with Ca^2+^ at biologically relevant concentrations was shown further increase in bacterial aggregation via cationic bridging. Negative Gibbs free energy (ΔG) values in iTC data confirmed that the interaction between DNA and Ca^2+^ is thermodynamically favourable and that the binding process is spontaneous and exothermic owing to its highly negative enthalpy. Removal of eDNA through DNase I treatment revealed that Ca^2+^ alone did not enhance cell aggregation and biofilm formation. This discovery signifies the importance of eDNA and concludes that existence of eDNA on bacterial cell surfaces is a key facilitator in binding of Ca^2+^ to eDNA thereby mediating bacterial aggregation and biofilm formation.

## Introduction

Biofilms play a crucial role in both medical and non-medical contexts such as harmful bacterial infections and biocorrosion [1], [2] and beneficial impacts in applications in bioremediation, bioelectricity production and wastewater treatment [3]–[5]. In most bacterial species, biofilm formation is mediated via bacterial self-produced matrix [6]. The matrix, which primarily consists of proteins, polysaccharides, lipids, RNA and extracellular DNA (eDNA) is involved in bacterial adhesion to surfaces and cell-to-cell attachment during the initial stages of biofilm formation and in the formation of microcolonies and ultimately biofilm maturation [7]. eDNA is a central biopolymer in matrix, released due to the various mechanisms such as, lysis of sub populations of bacterial cells mediated by prophages, lytic proteins, enzymes and metabolites such as phenazines [8]–[10] or by various physical and chemical treatments [11], [12] and also via active release by bacterial membrane vesicles/belbs [13]–[16]. eDNA plays an essential role in various stages of biofilm formation including initial bacterial adhesion, aggregation, microcolony formation, determining biofilm architecture [9], [17]–[19]. eDNA provides mechanical stability to biofilms and protects bacterial cells in biofilms from physical stress, antibiotics and detergents [7], [20], [21]. Recent studies showed that eDNA chelation of divalent cations induces genes involved in modification of bacterial cell surface properties that favour resistance of biofilms to antimicrobial agents and detergents [20]. Divalent cations such as Ca^2+^ stabilize bacterial cell walls [22] and promote ionic bridging between bacterial cells via interaction between negatively charged cell membranes and other biopolymers in matrix [22], [23]. At biologically relevant Ca^2+^ concentrations, 0.7 to 1.4 mM in blood serum [23] and 0.4 to 1.7 mM in environmental water samples [23], Ca^2+^ ions influence bacterial adhesion and biofilm formation [23]–[25]. It also acts as a sensory ion for gene expression of biofilm-associated growth [26].

Bacterial adhesion, aggregation and subsequent biofilm formation is influenced by the presence of various chemical compositions on bacterial cell surfaces. The presence of various molecules such as proteins and DNA determines surface properties including cell surface hydrophobicity (measured using contact angles). Hydrophobicity determines surface energies and subsequently influences the physico-chemical interactions between interacting bacterial cell surfaces. The physico-chemical interactions include non-specific attractive Lifshitz van der Waals (LW) interactions, attractive or repulsive electrostatic double layer interactions, and Lewis acid-base interactions and hydrophobic forces/interactions (based on electron-donating and accepting properties of surfaces) [27], and ionic bridging by divalent cations (Ca^2+^, Mg^2+^) [23], [24]. Recent reports suggest that the presence of eDNA on bacterial cell surfaces promotes surface hydrophobicity and influences attractive acid-base interactions and therewith promotes initial bacterial adhesion and aggregation [17], [18], [28]. DNA also binds divalent cations such as Ca^2+^ and undergoes major structural changes upon doing so [29].

Considering the importance of eDNA in bacterial biofilm formation and the interactions between Ca^2+^ and DNA we hypothesized that the combination of Ca^2+^ and eDNA promotes bacterial aggregation and subsequent biofilm formation. For this, we tested our hypothesis in aggregation and biofilm formation assays using a variety of Gram–negative (*Pseudomonas aeruginosa* PA14, *Aeromonas hydrophilla, Escherichia coli*) and Gram-positive (*Staphylococcus aureus*, *Staphylococcus epidermidis*, *Enterococcus faecalis*) bacterial species by adding exogenous Ca^2+^ and before and after removal of eDNA by DNase I treatment. The thermodynamics of Ca^2+^ binding with DNA was determined by using isothermal titration calorimetry (iTC) at constant temperature and pressure. iTC determines the physico-chemical interactions that drive many biological processes including binding of biopolymers with ions, folding of proteins and bacterial aggregation by measuring the changes in Gibbs energy (ΔG) based on changes in enthalpy (ΔH) and entropy (ΔS) when the interactions occur [30], [31]. During the interactions if heat is released from the system to the environment, then the ΔG is generally negative and the interaction is considered to be favourable, exothermic and spontaneous.

To this end *P. aeruginosa* PA14 has been chosen as a model organism to investigate the surface characteristics i.e. contact angle (measured using water, formamide and diiodomethane) and subsequent surface thermodynamics analysis in presence and absence of naturally occurring eDNA and added Ca^2+^ ions. The rationale/principle behind using polar liquids (e.g. water and formamide) was to determine electron accepting and electron donating parameters of acid-base interactions. This is because polar liquids/molecules have high surface tension/energy and tend to accept and donate electrons with interacting surfaces. In contrast the non-polar liquids (e.g. diiodomethane) have low surface energy and are not involved in electron donating or accepting parameters hence used for determining only Lifshitz-Van der Waals interactions.

To the best of our knowledge, the role of Ca^2+^ ions in eDNA mediated bacterial aggregation and biofilm formation has not yet been investigated. Therefore, the present work investigates for the first time the concurrent role of Ca^2+^ ions and eDNA in mediating aggregation and biofilm formation in divergent bacterial strains.

## Materials and Methods

### Bacterial species and culture conditions

Bacterial strains listed in table 1 (used in this study) were obtained from the culture collection of the Centre for Marine Bioinnovation (CMB), School of Biotechnology and Biomolecular Sciences, UNSW, Australia. The bacterial strains were grown aerobically in Luria Broth (LB) medium for 24 h at 37°C in a static incubator. After growth, strains were harvested by centrifugation at 5000×g for 5 min at 10°C. Where indicated, naturally occurring eDNA was removed from cell surfaces by pre-treating bacterial suspensions with 25 units of DNase I (Life technologies/Invitrogen) in the presence of 5 mM MgCl_2_ for 90 min at room temperature under static conditions and subsequently washed twice with phosphate buffered saline (PBS) (in order to remove any possible DNA debris, due to DNase I treatment) and finally resuspended in PBS.

**Table 1:** Bacterial strains used in this study and their sources.

Bacterial strains	Source or references
*P. aeruginosa* PA14 wildtype	[8]
A. *hydrophila*	[58]
*E. coli*	[58]
*S. aureus*	this study/CMB culture collection
*S. epidermidis*	this study/CMB culture collection
*E. faecalis*	[58]

### Quantification of bacterial growth/cell density

The optical density of *P. aeruginosa*, *A. hydrophilla, S. aureus* and *S. epidermidis* cultures (over 24 h at 37°C in a static incubator) in LB medium were recorded at various growth times (0, 4, 8, 18 and 24 h) using a Bio-Rad Smartspec 3000 (Bio-Rad Laboratories Pvt Ltd, USA) at 600 nm using plain LB medium without bacterial cells as blank.

### Comparison of live and dead cells in planktonic bacterial culture using fluorescence microscopy

*P. aeruginosa* and *S. aureus* (used as model strains) cultures grown in LB medium for 24 h at 37°C in a static incubator were used as is without harvesting/centrifugation to compare differences in dead cells. A 1 ml aliquot of the bacterial culture (averaging OD 2.3) was allowed to adhere on 6 well polystyrene plates (Costar, NY, USA) for 30 min at room temperature in static condition. After 30 min the well plate surfaces were gently washed with PBS once in order to remove any loosely bound bacterial cells (since loosely bound cells possibly interfere with microscopy imaging). The remaining adhered bacteria to the well plate's surface were imaged after staining with the Live/Dead stain (Invitrogen, Oregon, USA) for 15 min in the dark using fluorescence microscopy (Olympus DP71, Tokyo, Japan).

### eDNA concentration in bacterial cell free supernatant

After 24 h of bacterial growth and subsequent centrifugation, supernatants were separated from bacterial pellets by simply transferring to new tubes. In order to remove remaining bacteria, supernatants were filtered using 0.22 μm filters (Millipore). eDNA in filtered supernatants was quantified using the double stranded DNA fluorescent dye assay (dsDNABR) from Qubit, Invitrogen using their standard protocol. To quantify DNA the Qubit 2.0 fluorometer was calibrated using the standards (dsDNABR calibration standards 1 and 2) supplied by Qubit Invitrogen. After calibration, the total amount of dseDNA present in the supernatants was quantified directly by mixing 20 μl aliquots of supernatants with 180 μl dsDNABR assay buffer-fluorescent dye mixture in Qubit assay tubes, followed by vortexing and incubating for 2 mins at room temperature. The intensity of the fluorescent dye after binding with eDNA in the supernatant was measured by using the Qubit 2.0 fluorometer (Invitrogen, Life Technologies, CA, USA).

### Aggregation and settling of bacteria as a function of addition of Ca^2+^ in the presence and absence of eDNA

To study the aggregation pattern of all bacterial strains with different concentrations of added Ca^2+^ (as CaCl_2_.2H_2_O (Univar, NSW Australia)) (0, 10, 100, 1000 μM), in the presence and absence of eDNA, bacterial cells were first diluted to OD  = 0.5±0.05/ml in PBS. The percentage reduction in optical density (OD) resulting from bacterial cells settling due to aggregation in the presence and absence of eDNA and as a function of added Ca^2+^ concentration was monitored after 90 min incubation in 1 ml plastic cuvettes (SARSTEDT, Germany) at 600 nm using a spectrophotometer (Bio-Rad Smart Spec™ 3000). PBS without bacteria was used as a blank. For additional control experiments, bacterial cells were also treated with heat inactivated DNase I (heating DNase I for 30 min at 75°C) and subjected to the aggregation assay. Aggregation was quantified according to equation 1: X = 100× (OD1–OD2)/OD1 [28]. Where OD1 is the initial OD at the start of an experiment and OD2 is the OD after 90 min.

### Fluorescence microscopy imaging of *S. aureus and E. faecalis* adhesion and aggregation in the presence and absence of eDNA and added Ca^2+^

Initial adhesion and aggregation of planktonic bacterial cells in the presence and absence of naturally occurring eDNA and added Ca^2+^ were studied using fluorescence microscopy. This was done by suspending bacterial cells (with or without DNase I treatment) in PBS (OD  = 0.5±0.05/ml) as mentioned previously and then mixed (by vortexing) with added Ca^2+^ (0 and 1000 μM). A 1 ml aliquot of bacterial cells were immediately added to 6 well polystyrene plates and incubated for 90 min at room temperature in static condition. After 90 min the well plate's surfaces were gently washed with PBS. The adhered bacteria to the well plate's surface were imaged after staining with the Live/Dead stain as described above.

### Aggregation and settling of bacteria on addition of exogenous DNA and 1000 μM Ca^2+^

To quantify bacterial aggregation in the presence of exogenous DNA and Ca^2+^ (1000 μM), chromosomal DNA was extracted from one day old cultures of *P. aeruginosa* using standard protocols [18]. The extracted DNA was then dissolved in PBS and the DNA concentration was determined using a Nanodrop UV/Vis spectrophotometer. DNase I treated cells of all bacterial strains with a cell density of OD  = 0.5±0.05/ml in 900 μl PBS were mixed with 100 μl of a 1 μg/ml DNA solution or 100 μl PBS as a negative control in 1 ml plastic cuvettes by pipetting. The percentage settling of bacterial cells due to aggregation mediated by the presence or absence of exogenous DNA and addition of Ca^2+^ was calculated after 90 min using equation 1.

### Influence of DNase I treatment and addition of Ca^2+^ on biofilm formation

To quantify biofilm biomass, untreated and DNase I treated bacterial cells suspended in PBS (OD  = 0.5±0.05/ml) were incubated for 90 min in 96 well microtiter plates at 37°C. After 90 min of incubation wells were washed with PBS three times in order to remove any unattached bacterial cells. Subsequently, 200 μl of LB medium was added to the wells and incubated for 24 h at 37°C in order to initiate biofilm formation. After 24 h of biofilm growth, the LB medium was gently removed by pipetting and subsequently rinsed with PBS three times. The total biofilm biomass attached to the wells was quantified using O'Toole's crystal violet assay at 550 nm [32].

### eDNA release during biofilm growth

To establish the amount of eDNA released during biofilm growth, the biofilm biomass grown over 0, 16 and 24 h was suspended in 1 ml of PBS. The biomass suspended in PBS was then filtered with 0.22 μm filters to remove any bacterial cells and subsequently eDNA concentration present in filtered PBS (supernatants) was quantified as above.

### Contact angle measurements and surface thermodynamics of *P. aeruginosa* in the presence and absence of eDNA and added Ca^2+^

For contact angle measurements *P. aeruginosa* cultures were incubated for 90 min in the presence and absence of eDNA either as is (with no added Ca^2+^) or with addition of 1000 μM Ca^2+^. Bacterial lawns were prepared by depositing bacteria from suspension on a 0.2 μm nitrocellulose filter (Millipore) using negative pressure [33]. The filters were air dried at room temperature and contact angles were measured with most commonly used standard polar (water, formamide) and non-polar (diiodomethane) liquids using a goniometer (KSV model 200, KSV instrumentation Pvt. Ltd, Finland) following the sessile drop technique [33].

To conduct surface thermodynamical analysis, contact angles were converted into Lifshitz-Van der Waals (γ^LW^) and acid-base (γ^AB^) surface free energy components using the LW-AB approach [34], [35]. Subsequently, the acid-base component was separated into an electron-donating (γ^−^) and electron–accepting (γ^+^) parameter [34]. Different components of the surface energies were then further used to calculate the total interfacial free energy of aggregation (Total ΔG) at close contact as separated in a Lifshitz-Van der Waals (LW ΔG) and an acid-base (LW ΔG) part [35].

### DNA-Calcium binding/interaction

Isothermal Titration Calorimetry (iTC) was used to measure the binding affinity (Ka), enthalpy changes (ΔH), and binding stoichiometry (n) of the interaction between Ca^2+^ to DNA using a MicroCal iTC 200. DNA at a concentration of 50 ng/μl (measured using Nanodrop) or milliQ water as a negative control was mixed with CaCl_2_ (final concentration 1.2 mM) and divided into 25 injections, with an interval of 240 sec after each injection. All measurements were performed at 25°C, with a reference power of 5 µcal/sec and stirring speed of 800 rpm. Data was analysed using Origin 7.0 software (MicroCal). Gibbs free energy changes (ΔG) were determined using the relationship: ΔG  =  ΔH-TΔS where, ΔH is the enthalpy change, T is temperature (Kelvin) and ΔS is the change in entropy.

### Statistical analysis

The student's t-test was used for statistical analysis and differences were considered significant if *p*<0.05.

## Results

### Quantification of eDNA in supernatants of planktonic grown bacteria

**Figure 1:**
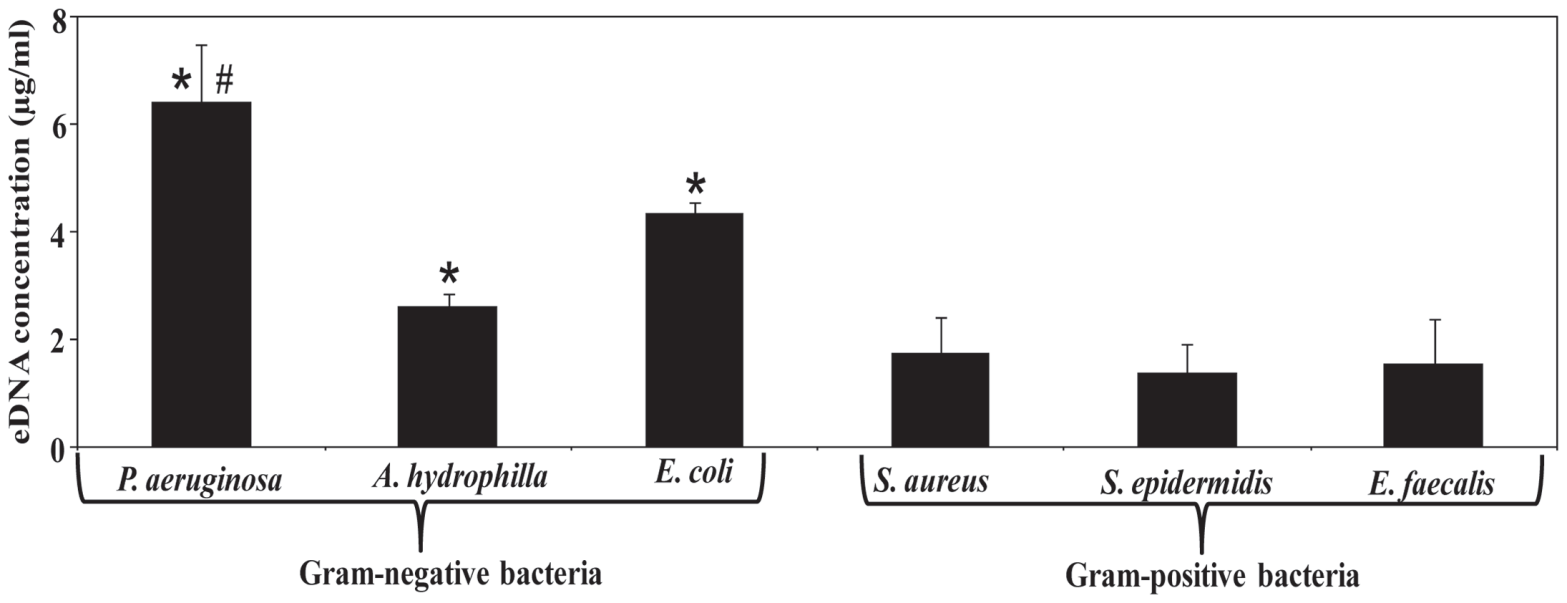
Quantification of eDNA in planktonic growth culture. Concentration of eDNA in supernatants of planktonic cultures of Gram-negative and Gram-positive bacterial strains grown for 24 h. Error bars represent standard deviations from multiple cultures (n = 4). Asterisks indicate statistically significant differences (*p*<0.05) in eDNA concentration in comparison to *S. aureus, S. epidermidis and E. faecalis* whereas, hash indicates *P. aeruginosa* supernatants has statistically significantly higher concentration of eDNA in comparison to all other bacterial strains.

Figure 1 shows that *P. aeruginosa* supernatant has the highest amount of eDNA (6.5 μg/ml) followed by *E. coli* (4.5 μg/ml) and *A. hydrophilla* (2.5 μg/ml). All Gram-positive (*S. aureus*, *S. epidermidis* and *E. faecalis*) strains showed significantly less eDNA in its supernatants averaging only 2 μg/ml. The optical density of bacterial cultures (*P. aeruginosa, A. hydrophila*, *S. aureus* and *S. epidermidis*) measured at various time intervals (0, 4, 8, 18 and 24 h) did not show any significant difference (Fig. S1A). However, fluorescence microscopy imaging revealed that planktonic cultures of *P. aeruginosa* have more dead cells in comparison to *S. aureus* (Fig. S1B).

### Influence of naturally occurring eDNA and addition of Ca^2+^ on bacterial aggregation

**Figure 2:**
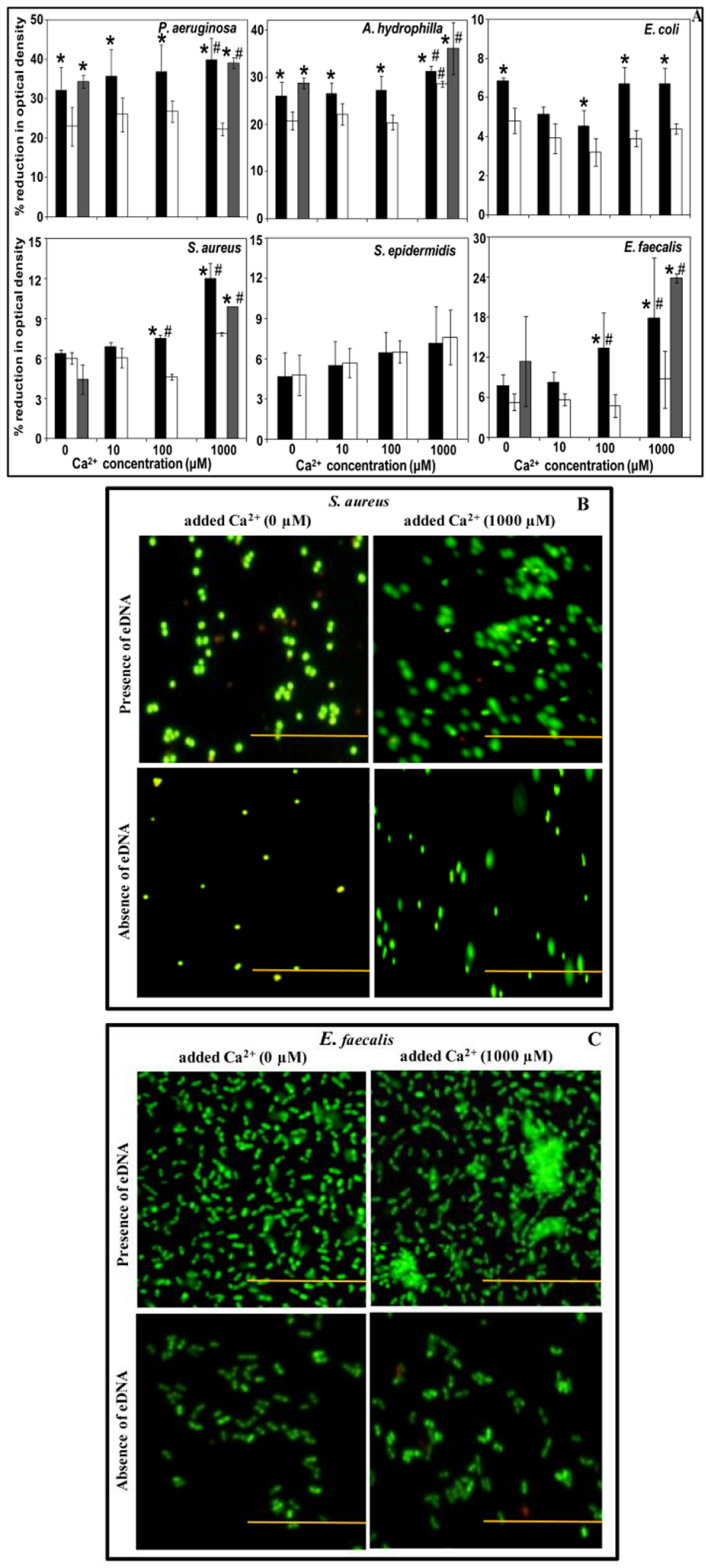
Influence of naturally occurring eDNA and added Ca^2+^ on bacterial aggregation. Percentage reduction in optical density of Gram-negative and Gram-positive bacteria in PBS, showing patterns of aggregation. The black, white and grey bars represent bacterial aggregation in presence of naturally occurring eDNA, absence of naturally occurring eDNA (DNase I treated) and heat inactivated DNase I treated respectively. Error bars represents standard deviations from multiple cultures (n = 5). Asterisks and hash indicate statistically significant differences (*p*<0.05) between data obtained in the presence (including heat inactivated DNase I) of eDNA in comparison to absence of eDNA and in presence and absence of Ca^2+^ respectively (A). Fluorescence microscopy imaging showing patterns of *S. aureus* and *E. faecalis* adhesion and aggregation in presence and absence of eDNA and added Ca^2+^ (1000 μM) on 6 polystyrene well plates surfaces after incubation for 90 min in room temperature under static condition (scale bar 50 μm) (B and C).

The settling of Gram-negative bacteria under gravity as a consequence of aggregation was influenced more by the presence of eDNA than aggregation of Gram-positive bacteria (Fig. 2A). Untreated, heat inactivated DNase I and DNase I treated bacterial cell preparations were incubated with added Ca^2+^ at different concentrations and assessed in aggregation assays. Figure 2A illustrates the role of Ca^2+^ in mediating bacterial aggregation in the presence (including results of heat inactivated DNase I) and absence of eDNA. In the absence of eDNA and added Ca^2+^ all three Gram-negative bacterial strains (*P. aeruginosa, A. hydrophila*, and *E. coli*) responded with a significant decreases in settling. In contrast, the Gram-positive bacteria (*S. aureus*, *S. epidermidis* and *E. faecalis*) showed no significant difference in aggregation. However, in the absence of eDNA, addition of Ca^2+^ (10–1000 μM) resulted in a significant reduction in aggregation for *P. aeruginosa*, *E. coli*, *S. aureus* and *E. faecalis* but not for *S. epidermidis*. At biologically relevant concentrations, Ca^2+^ (1000 μM) resulted in a significant increase in aggregation of *A. hydrophila* regardless of the presence of eDNA, however in presence of eDNA the aggregation was significantly higher than in its absence. Out of the six strains considered the aggregation of *S. epidermidis* was unique in that it was not influenced by the removal of eDNA.

The settling (aggregation) assay revealed that the presence of eDNA (including results of heat inactivated DNase I) promoted aggregation for all three Gram-negative bacterial strains used in this study (*P. aeruginosa*, *A. hydrophila* and *E. coli*) and two out of three Gram-positive bacteria tested (*S. aureus* and *E. faecalis*) regardless of the Ca^2+^ concentration. Further, addition of Ca^2+^, especially at 1000 μM, aggregation increased significantly, from 31% at 0 μM to 40% at 1000 μM for *P. aeruginosa*, 26% at 0 μM to 32% at 1000 μM Ca^2+^ for *A. hydrophila*, from 6% at 0 μM to 12% at 1000 μM Ca^2+^ for *S. aureus* and from 18% at 0 μM to 24% at 1000 μM Ca^2+^ for *E. faecalis* (Fig. 2A).

In the presence of naturally occurring eDNA and exogenously added Ca^2+^ (1000 μM) *S. aureus* and *E. faecalis* showed more adhesion and aggregation in comparison to the presence of eDNA alone. Interestingly, removal of eDNA by DNase I treatment significantly decreased *S. aureus* and *E. faecalis* adhesion and aggregation, regardless of added Ca^2+^ (1000 μM) (Fig. 2B and C).

### Influence of exogenous addition of DNA and Ca^2+^ on bacterial aggregation

**Figure 3:**
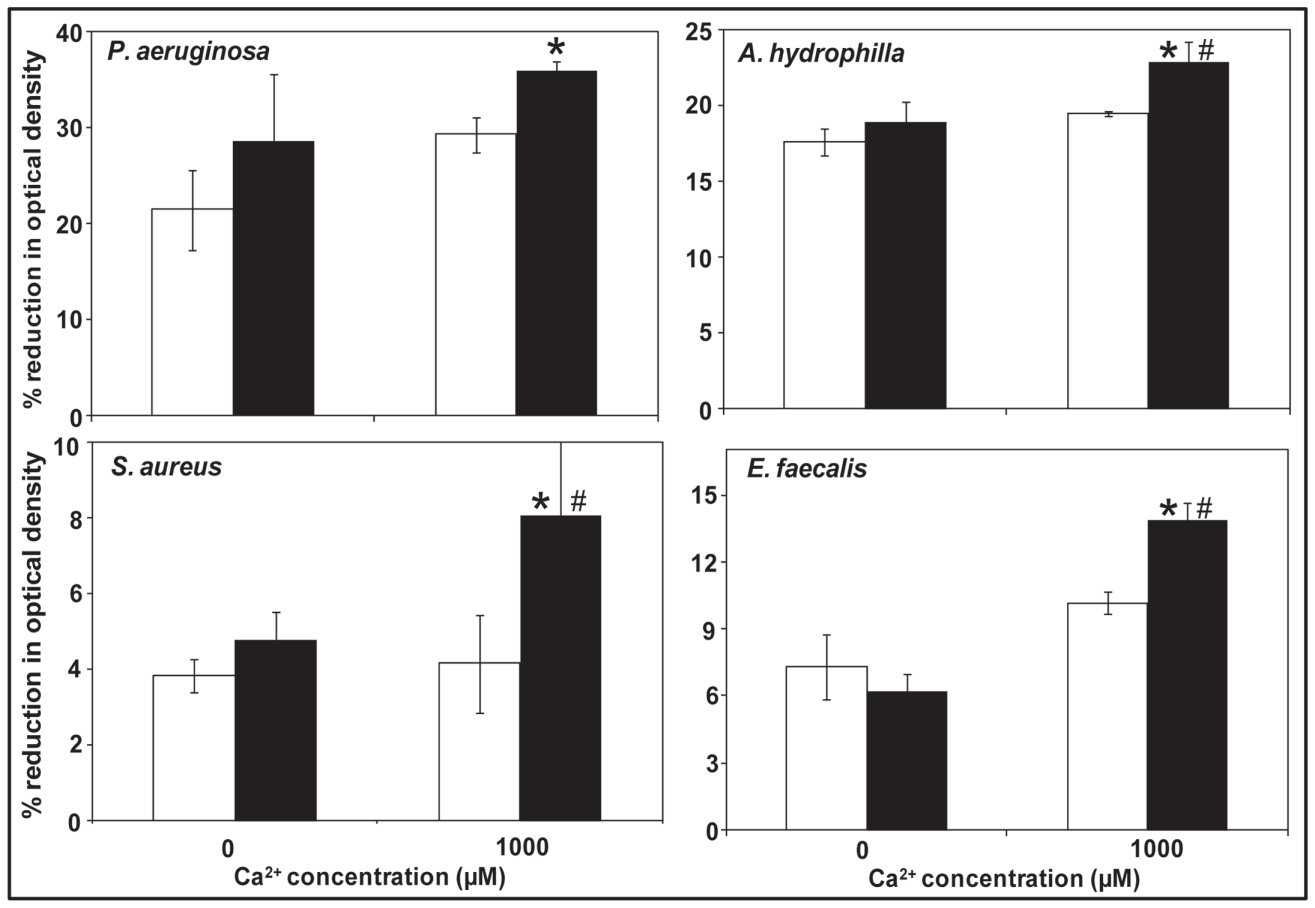
Influence of exogenous addition of DNA and Ca^2+^ on bacterial aggregation. Percentage reduction in optical density after 90^2+^ (1000 μM). Error bars represent standard deviations from multiple culture (n = 4). Asterisks and hash indicate statistically significant differences (*P*<0.05) between data obtained in the presence or absence of exogenous DNA and in presence and absence of Ca^2+^ respectively.

The influence of added Ca^2+^ and exogenous DNA on bacterial aggregation was further investigated by incubating DNase I pre-treated bacterial strains with and without addition of exogenous DNA (1 μg/ml) and Ca^2+^ (Fig. 3). After 90 min of incubation all four strains tested (*P. aeruginosa*, *A. hydrophilla*, *S. aureus* and *E. faecalis*) showed significant increases in bacterial aggregation in addition of both DNA and Ca^2+^ (1000 μM) in comparison to aggregation in addition of Ca^2+^ alone. Further, *A. hydrophilla*, *S. aureus* and *E. faecalis* showed significant increases in aggregation when both DNA and Ca^2+^ were added in comparison to aggregation observed in the addition of DNA alone (Fig. 3).

### Contact angle and surface thermodynamics of *P. aeruginosa* before and after DNase I treatment and addition of Ca^2+^

Table 2 shows the changes in contact angle and surface energy values of *P. aeruginosa* in the presence and absence of eDNA and added Ca^2+^. In the presence of eDNA *P. aeruginosa* lawns gave a higher water contact angle of about 50°, indicating that the bacterial cell surfaces are more hydrophobic in the presence of eDNA than in its absence (contact angle about 30°). However, addition of Ca^2+^ did not influence the contact angle values aka hydrophobicity of *P. aeruginosa* cell surface regardless of the presence or absence of eDNA. Hydrophobicity determines surface energies (potential of any surface to bind with other surface or liquids/molecules through disruption/formation of bonds). The surface energy components: Lifshitz-Van der Waals (arises from entire bacterial cell body) which is generally attractive (due to dipole interactions between molecules or between parts of same molecules) values about 35 mJ/m^2^ were not affected by the presence or absence of eDNA or added Ca^2+^. However, the electron donating and electron accepting parameters of the acid-base component (arising from specific molecules present on bacterial cell surfaces) that form the basis for the surface hydrophobicity of bacterial cells was significantly different in the absence of eDNA. Especially, in presence of eDNA the electron donating group has surface energies about half the value (averaging 25 mJ/m^2^) than in absence of eDNA, regardless of added Ca^2+^. This result indicates that in the presence of eDNA the bacterial cell surface has low surface energies but strong hydrophobic forces that promote aggregation.

**Table 2:** Contact angle and surface free energy components of *P. aeruginosa* PA14 before and after DNase I Treatment and addition of Ca^2+^.

Bacterial strain	Added Ca^2+^ conc. (μM)	Contact angle (θ) (degrees)			Surface energy components (mJ/m^2^)			
		Water	Formamide	Diiodomethane	*γ* ^LW^	*γ* ^_^	*γ* ^+^	*γ* ^AB^
*P.aeruginosa* PA14	**Presence of eDNA**							
	0	**51.6±5.7**	38.8±1.3	50.0±1.0	34.2±0.6	**25.9±4.3**	**1.4±0.2**	11.9±0.9
	1000	**50.4±7.4**	40.0±2.8	48.3±1.1	35.1±0.7	**27.7±5.2**	**0.9±0.2**	10.2±0.3
	**Absence of eDNA**							
	0	31.0±1.8	36.0±4.0	45.5±6.3	36.7±2.4	52.1±4.9	0.4±0.4	8.4±5.2
	1000	33.6±2.9	40.3±4.6	48.5±2.1	35.1±0.8	53.0±5.4	0.3±0.2	7.0±3.4

Measurement of contact angles on *P. aeruginosa* PA14 lawns with water, diiodo-methane and formamide and subsequent surface free energy components: Lifshitz-Van der Waals component (*γ* ^LW^), electron-donor (*γ* ^_^) and acceptor (*γ* ^+^) for the acid-base component (*γ* ^AB^) are derived from measured contact angle in the presence and absence of eDNA and Ca^2+^ (1000 μM). ± represents standard deviations from the mean (n = 4). Data in bold indicate statistically significant (p<0.05) differences between presence and absence of eDNA.

### Interfacial free energy of aggregation of *P. aeruginosa* wildtype

**Figure 4:**
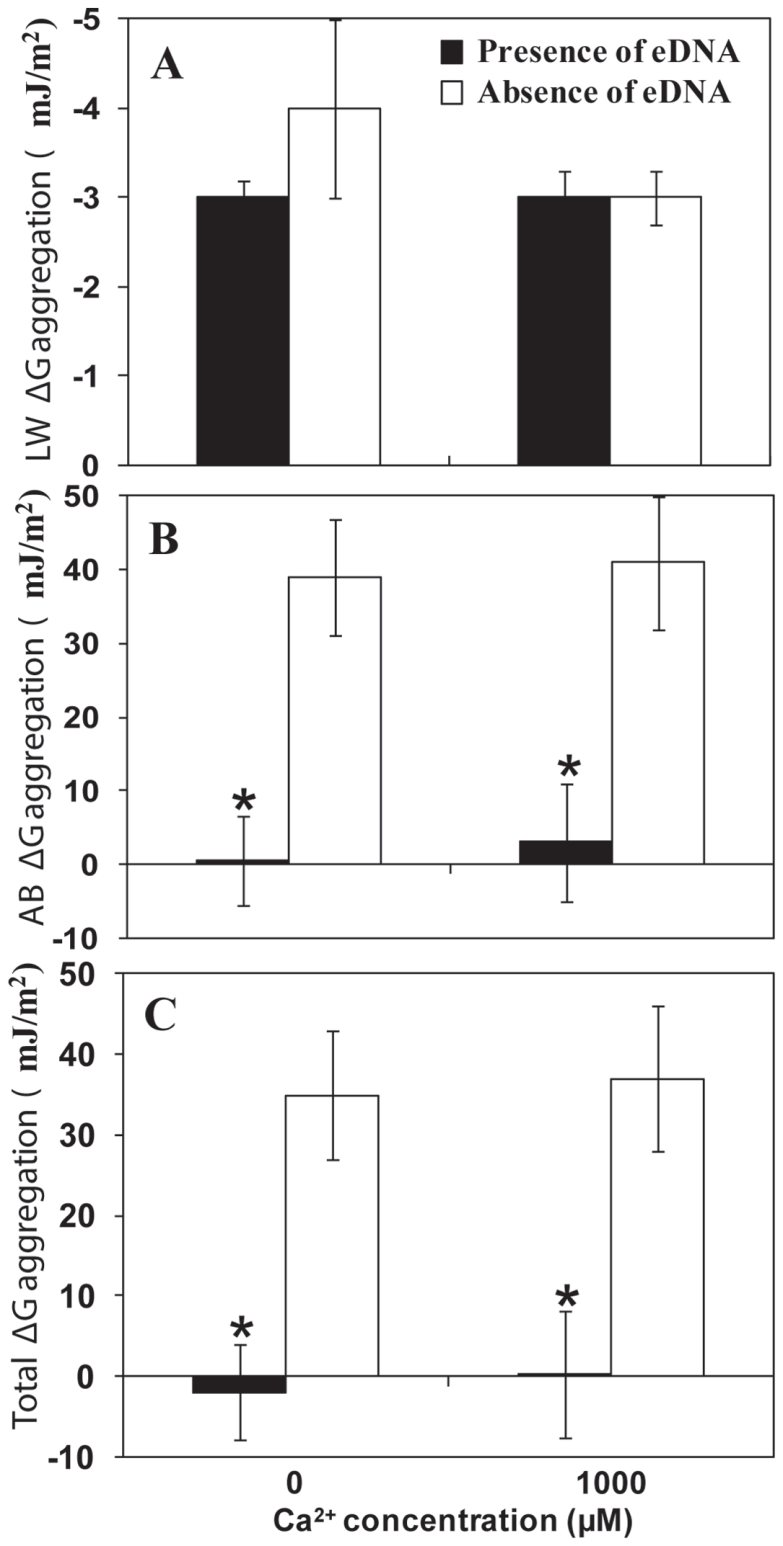
Interfacial free energy of aggregation of *P. aeruginosa* wildtype. The interfacial free energy of aggregation in the presence and absence of eDNA and Ca^2+^ (1000 μM) includes components: Lifshitz-Van der Waals (LW ΔG) (A) and acid-base (AB ΔG) (B) and total interfacial free energy (Total ΔG) (C) of aggregation of PA14. Error bars represents standard deviations from the mean (n = 4). Asterisks indicate statistically significant (p<0.05) differences in the free energy of aggregation in comparison to absence of naturally occurring eDNA, regardless of presence of Ca^2+^.

Figure 4 shows the changes in interfacial aggregation energies of *P. aeruginosa* at close approach in the presence and absence of eDNA and added Ca^2+^. Lifshitz-Van der Waals aggregation energies were negative averaging −3 mJ/m^2^ regardless of the presence of eDNA or Ca^2+^ (Fig. 4A). However, in the presence of eDNA acid-base aggregation energies were very low ranging from 0.5 at 0 μM Ca^2+^ to 3 mJ/m^2^ at 1000 μM Ca^2+^ and much higher (approximately 40 mJ/m^2^) in the absence of eDNA regardless of addition of Ca^2+^ (Fig. 4B). The total aggregation energies (a combination of Lifshitz-Van der Waals and acid-base aggregation energies) were close to zero (−2 at 0 μM Ca^2+^ to 0.5 mJ/m^2^ at 1000 μM Ca^2+^) in the presence of eDNA, whereas in the absence of eDNA the total aggregation energies remained high (approximately 35 mJ/m^2^) regardless of addition of Ca^2+^ (Fig. 4C). In brief, these results indicate aggregation in *P. aeruginosa* is a consequence of coordination between hydrophobic forces and acid-base interactions driven by the presence of specific surface molecules (eDNA).

### Thermodynamic of binding of Ca^2+^ with DNA

**Figure 5:**
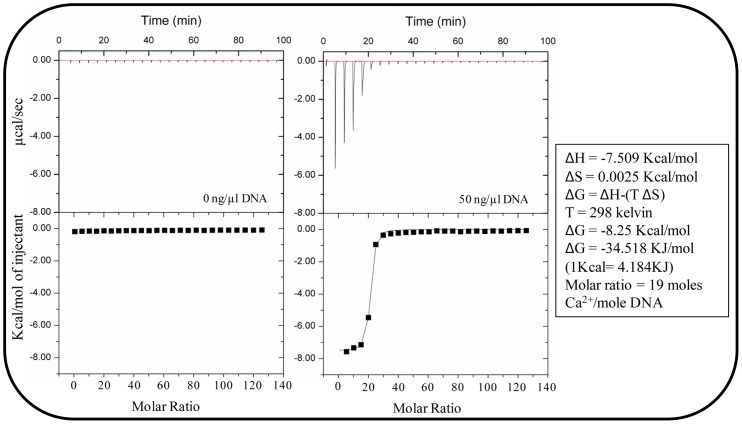
Thermodynamic of binding of Ca^2+^ with DNA. Isothermal titration calorimetry (iTC) studies to evaluate the interaction between DNA and Ca^2+^. Upper panel: Raw data for the titration of total 200 μl DNA (50 ng/μl) with total 1200 μM Ca^2+^. Lower panel: Integrated, dilution-corrected and concentration normalized titration data of the DNA with Ca^2+^. Data were fitted with the “One binding site model” of the Origin 7.0 data analysis software (MicroCal) with derived thermodynamic parameters including enthalpy (ΔH), entropy (ΔS) and Gibbs free energy (ΔG) and showing number of moles of Ca^2+^ binding to per mole of DNA at 25°C.

Figure 5 shows isothermal calorimetry analysis of the DNA-calcium interaction. The results show a large increase in Kcal/mol in the presence of DNA as Ca^2+^ was introduced exogenously. In the absence of DNA the Kcal/mol of Ca^2+^ injection remained static regardless of the number of injections. The results indicate that approximately 19 moles of Ca^2+^ interacts with 1 mole of DNA. The change in Gibbs free energy (ΔG) for the interaction is −8.25 Kcal/mol, composed of change in enthalpy (ΔH) with a value of −7.509 Kcal/mol and change in entropy (ΔS) at almost zero (0.0025 Kcal/mol) at constant temperature (298 Kelvin).

### Influence of added Ca^2+^ in biofilm formation before and after DNase I treatment and eDNA concentrations during biofilm growth

**Figure 6:**
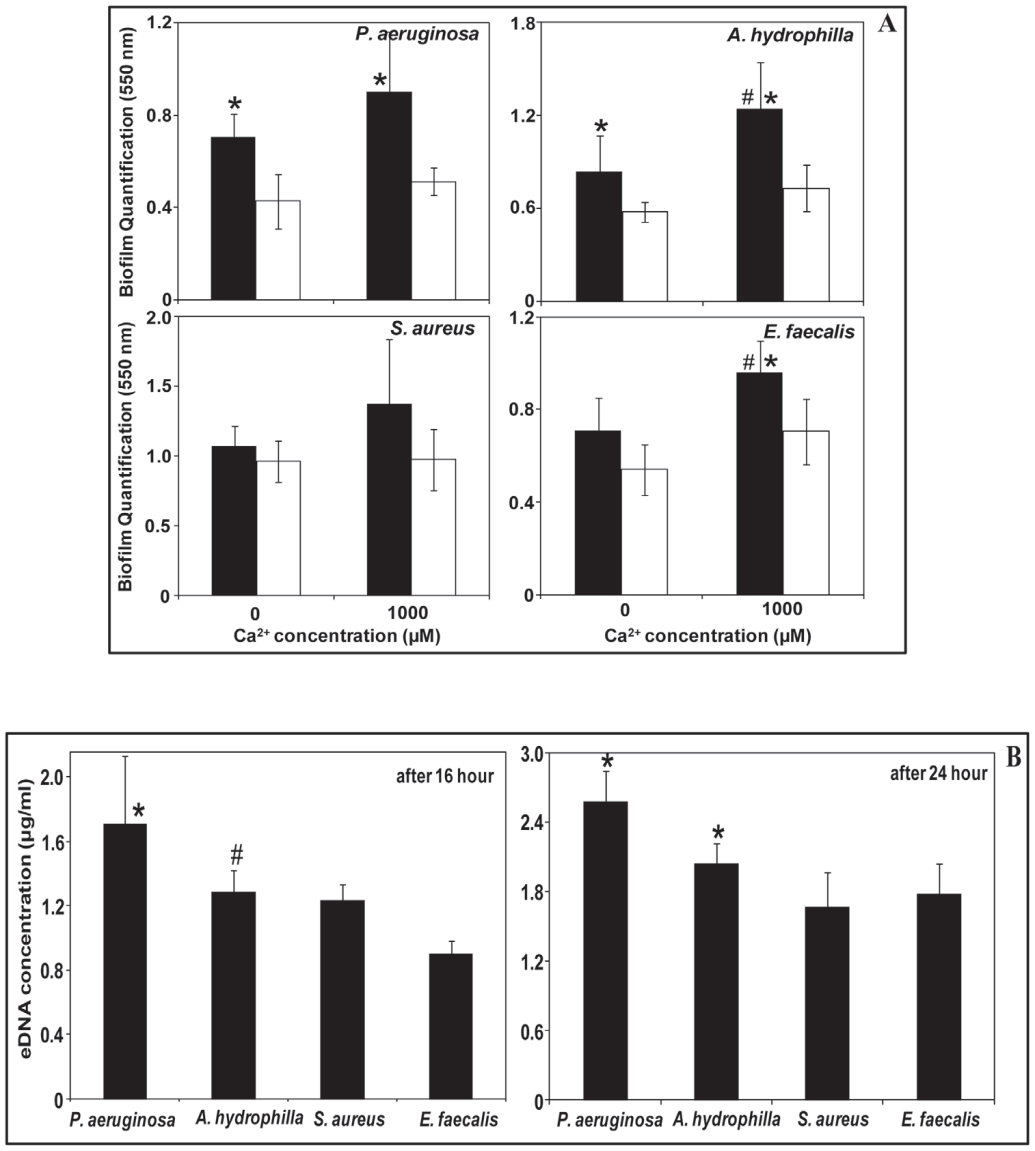
Influence of Ca^2+^ in biofilm formation before and after DNase I treatment and eDNA concentration during biofilm growth. Biofilm biomass quantification over 24(black) or absence (white) of naturally occurring eDNA (A). Error bars represents standard deviations from multiple cultures (n = 5). Asterisks and hash indicate statistically significant (*P*<0.05) differences between data obtained in the presence or absence of eDNA and in presence or absence of Ca^2+^ respectively. Concentration of eDNA at different growth time of biofilm formation for Gram-negative and Gram-positive bacterial strains (B). Error bars represent standard deviations from multiple cultures (n = 4). Asterisks indicate statistically significant (*p*<0.05) differences in eDNA concentration in comparison to both *S. aureus* and *E. faecalis*. Hash indicates the difference is statistically significant only in comparison to *E. faecalis*.

Figure 6A shows that eDNA enhances total biofilm biomass of Gram-negative bacteria. Addition of Ca^2+^ further increased biofilm biomass in *A. hydrophila* and *E. faecalis* over 24 h. In the absence of eDNA Ca^2+^ does not enhance biofilm biomass in any of the strains used in this study. Further, figure 6B shows concentration of eDNA increases with the increase in biofilm growth time and is higher in Gram-negative bacteria than Gram-positive bacteria.

## Discussion

Recent discoveries reported that eDNA bind with various biopolymers (polysaccharides, proteins) and metabolites (phenazines) produced by bacteria [21], [36], [37] and provides structural integrity to bacterial self produced matrix and biofilm promotion and stability. Likewise DNA, which is a negatively charged molecule, binds with positively charged Ca^2+^ via electrostatic interactions [38]. In biofilm biology, divalent cations like Ca^2+^ are primarily known for stabilizing bacterial cell walls and promoting ionic cross bridging among bacterial cells. In this study it is shown that Ca^2+^ binding with eDNA facilitates bacterial cell-to-cell aggregation and subsequent biofilm formation.

The significant increase in eDNA recorded in the supernatant of *P. aeruginosa* in comparison to all other bacterial strains tested is likely due to a combination of the active release of eDNA via membrane vesicles/blebs [13], [14] and through cell lysis mediated by pyocyanin [8]. No significant differences in kinetics of *P. aeruginosa* cell growth/density (OD) were observed in comparison to other bacterial strains and none of the strains used in this study are known to possess lytic proteins/enzymes. In contrast, microscopy imaging revealed an increase in pyocyanin mediated cell lysis and ultimately increased release of eDNA in *P. aeruginosa* cultures [8]. However, the involvement of active release of eDNA by membrane vesicles in *Pseudomonas* species cannot be ruled out. Variations in the concentration of eDNA between strains likely explain the differences in aggregation and biofilm formation behaviour between the bacteria used in this study. Gram-negative bacterial strains showed significant decreases in aggregation when eDNA was removed through DNase I treatment. In the presence of biologically relevant Ca^2+^ concentrations Gram-positive bacteria showed significant increases in aggregation. Interestingly, under conditions when both eDNA and Ca^2+^ are present an increase in bacterial aggregation was observed for 4 out of 6 bacterial strains and this increase is likely due to cationic bridging interactions mediated by Ca^2+^ [23], [24]. Microscopic evidence also revealed that eDNA increased bacterial adhesion to surfaces and Ca^2+^ promotes aggregation or clustering of bacteria. Increased bacterial adhesion to any kind of substratum surfaces by eDNA is due to strong interaction/adhesion forces and mature bond formation between bacterial cell surfaces in the presence of eDNA and substratum surfaces triggered by physico-chemical forces [17], [39].

Apart from cationic bridging interactions mediated by Ca^2+^ there are other influential factors that promote bacterial aggregation such as acid-base interactions. To investigate how acid-base interactions and cationic bridging complement each other in promoting bacterial aggregation we chose *P. aeruginosa* as model organism because of its naturally high eDNA content in its supernatant. In the presence of eDNA *P. aeruginosa* showed significant increases in cell surface hydrophobicity and DNA being a hydrophilic molecule likely interacts with hydrophilic (polar) sites on the *P. aeruginosa* cell surface, leaving hydrophobic (non-polar) sites unattended, consequently promoting hydrophobicity [18]. Surface thermodynamics analysis revealed that acid-base interactions and total interaction energies are more favourable for bacterial aggregation in the presence of eDNA when the free energy values are close to zero or negative. However, on hydrophobic or low surface energy surfaces hydrophobic forces that are the basis of acid-base interactions, play an influential role in promoting aggregation by repelling water and attracting adjacent bacteria. Small positive values represent an extremely low energy barrier that microbial cells can overcome due to the presence of biopolymers such as eDNA and cell appendages that extend hundreds of nanometers from the cell surface [40]. In support of this, removal of eDNA resulted in large increases (more than 50 fold) in the positive values in acid-base and total interaction energies signify unfavourable for bacterial aggregation.

Interestingly, incubation of bacterial cells with Ca^2+^ did not influence acid-base interactions energies regardless of the presence of eDNA, however, Ca^2+^ influenced bacterial aggregation. This indicates that Ca^2+^ binding with eDNA promotes cationic bridging only. Negative ΔG values in iTC data confirmed the interaction between DNA and Ca^2+^ and revealed that it is thermodynamically favourable and the binding process is spontaneous and exothermic (heat released during interaction) owing to its overall highly negative enthalpy. A previous calorimetric study reported that the presence of polypeptides (antigen I/II) on streptococcal cell surfaces promotes highly negative enthalpy associated with high heat release when binding with salivary protein [41]. Another iTC study showed higher negative enthalpy when an *S. aureus* strain with fibronectin binding protein on its cell surface binds to fibronectin in comparison to its mutant strain deficient in fibronectin binding protein [42]. Coaggregation between bacterial cells has been described as a consequence of interactions between various molecules on bacterial cell surfaces [31]. Our results in conjunction with previously published work using iTC on bacterial binding with macromolecules and aggregation suggests that negatively charged DNA molecules on bacterial cell surfaces will readily bind Ca^2+^ and promote aggregation.

**Figure 7:**
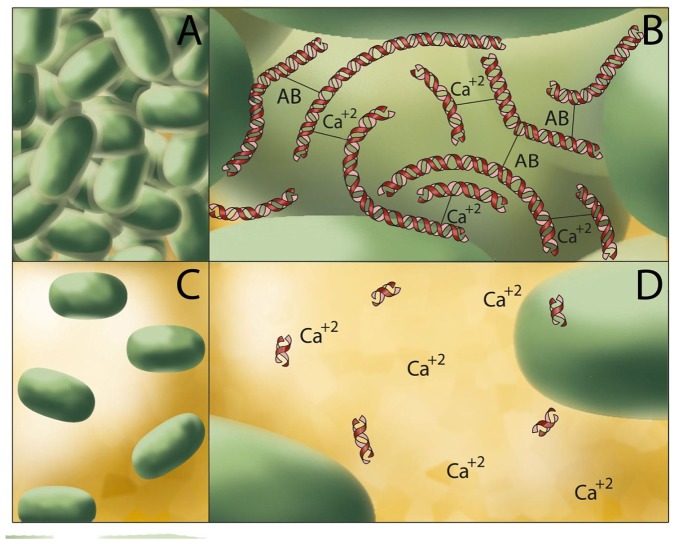
eDNA mediated bacterial aggregation via acid-base interactions and Ca^2+^ assisted cationic bridging. Schematic representation showing removal of eDNA influence acid-base interactions, and Ca^2+^ mediated cationic bridging between bacterial cells (B, D) and consequently bacterial aggregation (A, C).

Another study suggests that since most cells are negatively charged, including bacteria, adsorption of Ca^2+^ on bacterial cell surface decreases electrostatic repulsion and therefore facilitates adhesion and aggregation [31], [43]. This also likely explains why in presence of eDNA and Ca^2+^ bacteria adhered and aggregated more. At the bacterial cell surface, specific interactions could result from the strong affinity of Ca^2+^ at multiple functional sites. Ca^2+^ binds to several constituents of matrix including lipopolysaccharides [44], polysaccharides [45], [46] and teichoic acid [47] enhancing ionic bridging between bacterial cells by the formation of bridges between matrix/biopolymers sites having high affinity for divalent cations. Since biofilm matrix can represent up to 90% of the dry biofilm biomass [7] and eDNA is crucial for structural integrity of matrix [37], [48] and biofilm integrity [19], removal of eDNA likely plays a role in the structural disintegration of matrix. It should be noted that other biopolymers including polysaccharides, proteins or RNA could influence the structural integrity of biofilm matrices and bind with Ca^2+^ (via electrostatic interactions) affecting aggregation in a variety of bacterial strains. Studies comparing other biopolymers and eDNA in facilitating matrix integrity and aggregation through Ca^2+^ binding have not been undertaken. The results of the current study support the assertion that eDNA plays a central role in promoting Ca^2+^ regulated ionic cross-bridging and subsequent aggregation in most of the bacterial strains used in this study. Figure 7 illustrates for clarity how it is envisaged that removal of eDNA disrupts bacterial matrix, affects acid-base interactions and ionic-bridging and subsequent bacterial cell-to-cell interactions (aggregation). For *S. epidermidis* aggregation is not influenced by removal of eDNA by DNase I treatment is a clear indication that eDNA did not influence cell-to-cell interaction or matrix integrity. This is not surprising because it is well documented that in many *S. epidermidis* strains matrix formation, bacterial interactions and biofilm formation is primarily dependent on polysaccharides [49], [50]. *S. epidermidis*, which is well known for causing biomaterial-associated infection in humans produces different types of extracellular polysaccharides that are responsible for various roles ranging from bacterial adhesion to biofilm formation and promoting virulence [51], [52].

Ca^2+^ is known to increase the production of extracellular components such as proteins [26], and also induces virulence factors by increasing pyocyanin production in *P. aeruginosa* biofilm [53]. This observation is possibly due to modulation of genes influencing excess secretion of extracellular protein and pyocyanin and at the same time accumulation/trapping secreted proteins and pyocyanin in a well integrated or cross bridged Ca^2+^-eDNA-polysaccharides matrix. In contrast, Ca^2+^ does not influence release of eDNA under planktonic or biofilm growth conditions (Fig. S2) but promotes bacterial aggregation and subsequent biofilm formation. The biofilm consists of up to 90% of biopolymers constituent matrix [7] and only a fraction of it anchored to bacterial cell walls. The rest of the biopolymers including eDNA available or bound with other matrix constituents in biofilms plays an important role in modulating viscoelastic properties of biofilms [54]. Viscosity influences the ‘sticky’ character of biofilm matrix/slime [13] and further promotes mechanical strength and protection of biofilms from antibiotics and shear stress. The release of eDNA into planktonic culture supernatant increases viscosity and may provide protection from grazing or biocides. It could also promote transformation through DNA uptake and represents an excellent source of nutrition. Ca^2+^ has been recently discovered to prevent interactions of antimicrobial agents such as quaternary ammonium compounds with bacterial cell membrane [55] similarly eDNA impede antibiotics (aminoglycosides) mediated bacterial cell lysis [56].

The knowledge gained in this study on the role of eDNA/Ca^2+^ interactions in bacterial aggregation and biofilm development and previous reports published on eDNA in development of biofilms and the protection of cells confirms that eDNA triggers strong biofilm development. Hence in this era where efficacy of various antibiotics and antibacterial agents are fading DNase I based therapy could lead to efficient improvement in inhibition or treatment of various bacterial infections. DNase I which is already in use as an inhaler in the treatment of cystic fibrosis [57] could also be extended in other clinical applications. This includes engineering combinations of antibiotics with DNase I coatings on biomedical implants, or design of DNase I based wound care gels. Inclusion of DNase I in therapy will drastically reduce the initial bacterial adhesion, matrix formation and strong biofilm formation allowing the effective killing of remaining bacterial cells with the minimal use of antibacterial agents.

## Supporting Information

Figure S1 **Quantification of bacterial cell growth/density and fluorescence microscopy imaging of live and dead cells in planktonic culture.** Kinetics of growth of bacterial (*P. aeruginosa, A. hydrophila*, *S. aureus* and *S. epidermidis*) cell density (OD) in planktonic condition in LB medium measured at various time intervals (0, 4, 8, 18 and 24 h) (A). Comparison of live and dead cells in planktonic culture of *P. aeruginosa* and *S. aureus* grown in LB medium for 24 h at 37°C in a static incubator using fluorescence microscopy (scale bar 50 μm) (B).
(TIF)

Figure S2 **Quantification of eDNA concentration as a function of added Ca^2+^ concentration.** eDNA quantified in planktonic culture of *P. aeruginosa* grown in LB medium for 24 h at 37°C in a static incubator in presence of added Ca^2+^ (0, 1000 and 5000 μM).
(TIF)
