# Supplementary figures and images for: Influence of Calcium in Extracellular DNA Mediated Bacterial Aggregation and Biofilm Formation
Source: PLoS One. 2014 Mar 20;9(3):e91935. doi: 10.1371/journal.pone.0091935 (PMC3961253; doi:10.1371/journal.pone.0091935)

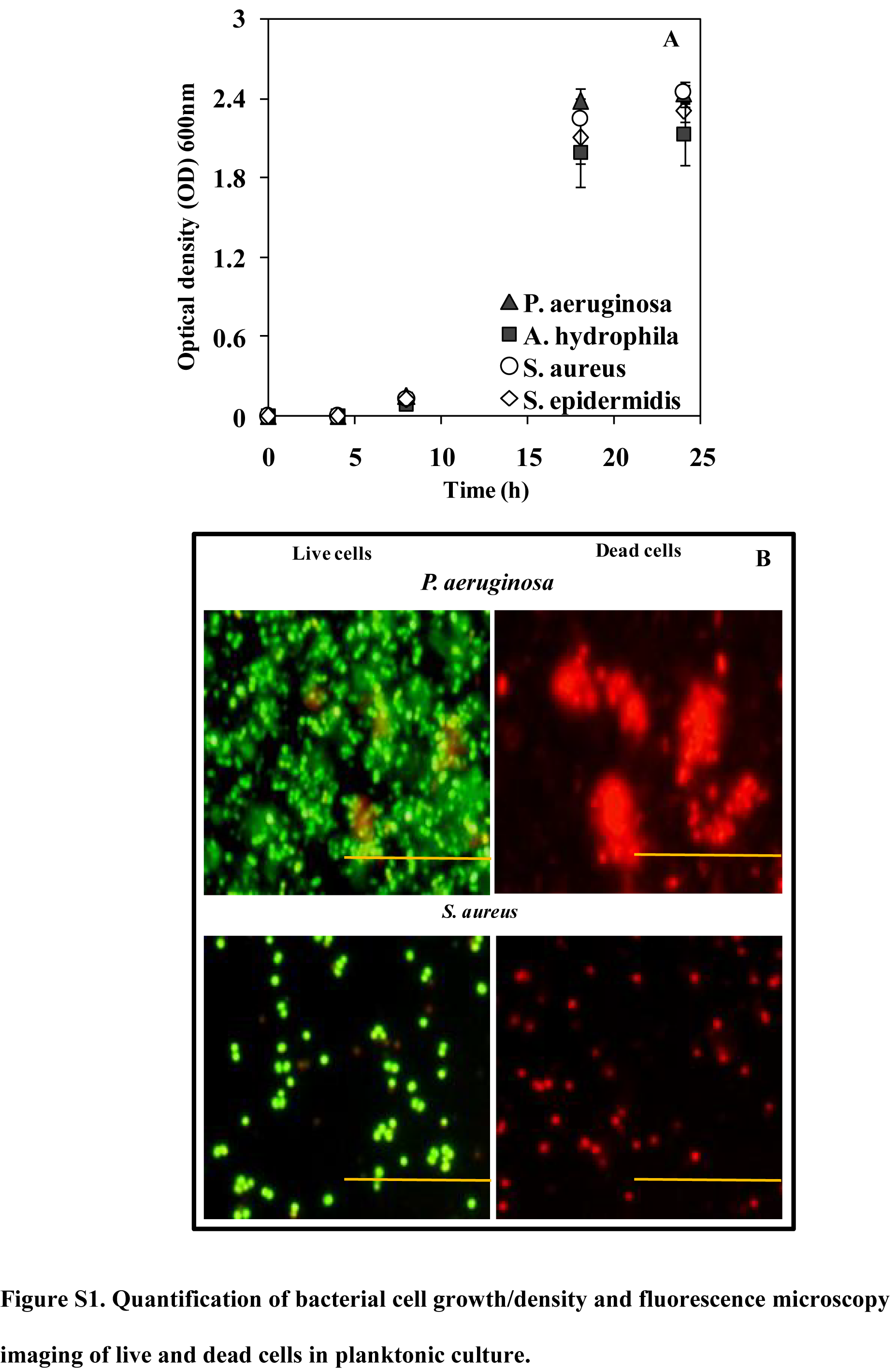

Supplement: Figure S1 — Quantification of bacterial cell growth/density and fluorescence microscopy imaging of live and dead cells in planktonic culture. Kinetics of growth of bacterial (P. aeruginosa, A. hydrophila, S. aureus and S. epidermidis) cell density (OD) in planktonic condition in LB medium measured at various time intervals (0, 4, 8, 18 and 24 h) (A). Comparison of live and dead cells in planktonic culture of P. aeruginosa and S. aureus grown in LB medium for 24 h at 37°C in a static incubator using fluorescence microscopy (scale bar 50 μm) (B). (TIF) [file pone.0091935.s001.tif]

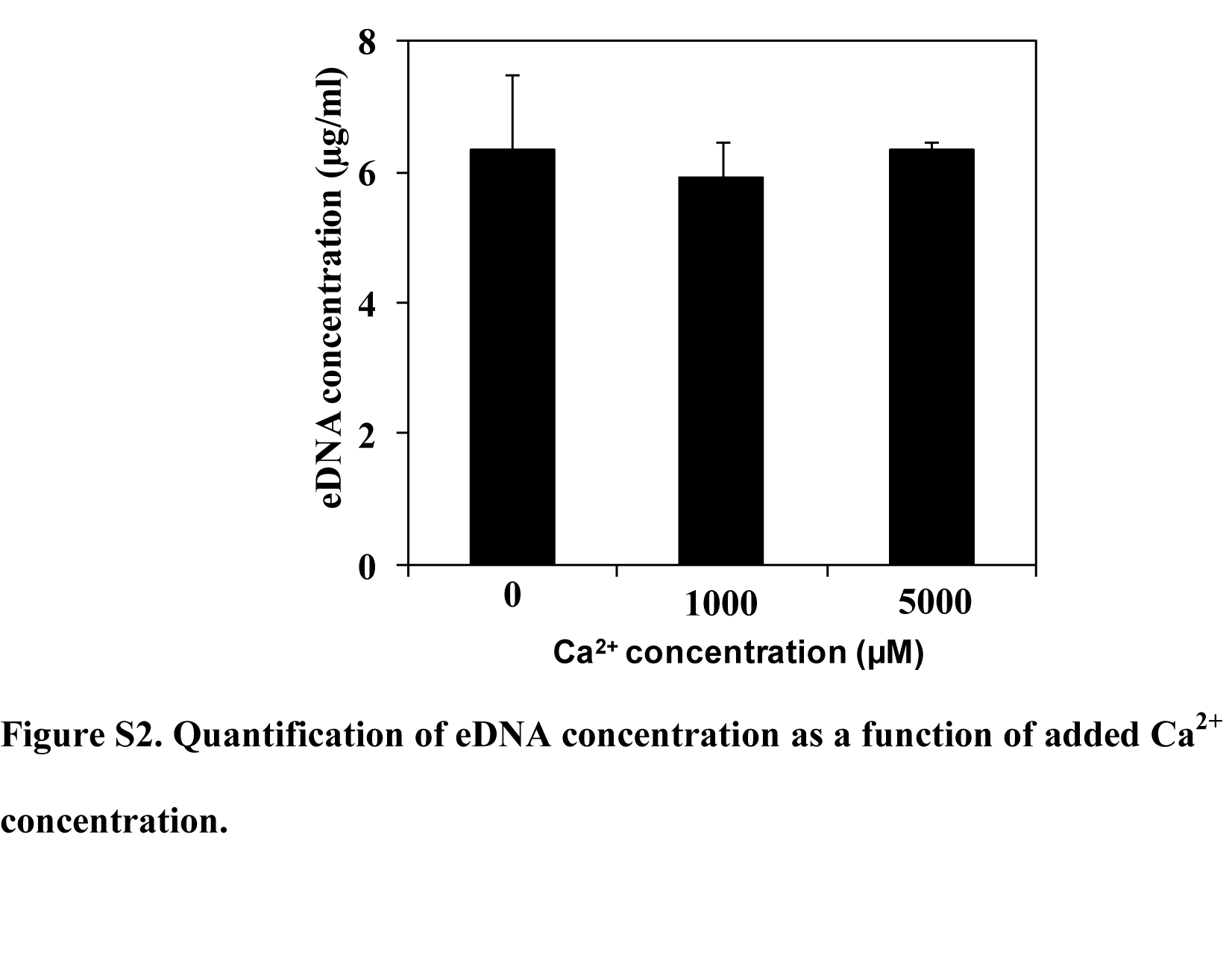

Supplement: Figure S2 — Quantification of eDNA concentration as a function of added Ca2+ concentration. eDNA quantified in planktonic culture of P. aeruginosa grown in LB medium for 24 h at 37°C in a static incubator in presence of added Ca2+ (0, 1000 and 5000 μM). (TIF) [file pone.0091935.s002.tif]
